# The Repetition of the Dose (Proceedings of the Brooklyn Hahnemannian Union)

**Published:** 1896-06

**Authors:** E. L. Close

**Affiliations:** Secretary


### "THE REPETITION OF THE DOSE."

(Proceedings of the Brooklyn Hahnemannian Union.)

The regular meeting of the Brooklyn Hahnemannian Union was held on February 29th, Dr. Baylies presiding, according to appointment. Dr. Fincke read a translation of an editorial by Dr. Villiers, of Dresden, comparative of different methods of practice, showing that, while the homœopathic law is the only one which satisfies, yet its progress largely depends upon the character of its representatives. Dr. Lutze spoke of the three provings of Anti-toxine recently made by the Kings County Medical Society, the value of which was rendered void because of the Carbolic-acid in which the Anti-toxine was preserved, nearly all the symptoms elicited being Carbolic-acid symptoms. Dr. Baylies added that there were no symptoms from the 1st decimal, but the 6th and 30th were followed by diarrhœa and tonsillitis, and the latter was cured with Belladonna. These three provings cost the society seventy-five dollars. Dr. Lutze spoke of the progress of Allopathy as guided by fad, which seemed to follow a curve and was now going back to the old method of using parts of the human body, or isopathy. He preferred potentiation by alcohol to that of animal propagation. Dr. John Campbell referred to the recent idea of tuberculized chickens.

At this point, the members having all assembled, the minutes of the previous meeting were read and accepted. Dr. Baylies' paper on "The Repetition of the Dose" was then read.

Dr. Baylies said in part that the object of the homœopathic prescription is cure, and to accomplish this there is more to be considered than merely symptom-similarity. The remedy to prove curative, often, must have attained a greater vibratile tension than can be reached in ordinary potencies, in order to harmonize with the perturbed vital dynamis. The high potency must be managed with greater care than the low.

The necessity of frequency of repetition of doses depends upon the activity of the individual medicine, the duration of its action, and the susceptibility of the patient, which at present can be determined only by experiment. The 200ths, in sthenic conditions, such as inflammation or fever, act well in doses repeated every two or three hours. In chronic diseases, or acute inflammations complicated with psora, the best results have been obtained from the single dose of the high potency—generally under CM. In diphtheria give the single dose, high, and repeat at cessation of improvement. Aggravation or suspension of improvement often follows repetition of high potencies. In grave forms of disease, like diphtheria, it is necessary to economize the medicinal force, in order to economize the vital force. Dr. P. P. Wells thought that a lower potency cannot well follow a higher.

In several cases of chronic diffused eczema much increased congestion of the skin followed administration of second dose after lapse of several weeks. The patient was cured, but recovery was retarded by the repetition.

Dr. Fincke said that, although there is great diversity of opinion on the subject there is uniformity in saying, if improvement continues, let the medicine act. Hahnemann himself has given different views, and we read many contradictions in the journals, as when one authority cautions *not* to repeat Lachesis, while another says it is one of the shortest-acting remedies.

Dr. Lutze spoke of a case of sleeplessness which had continued since an attack of scarlet fever. The man had the appearance of the fever again with grip. Belladonna cured him, but a repeated dose produced an aggravation.

Dr. Alice Campbell insisted that there ought to be some law. Dr. Fincke questioned if repetition could spoil the case. Some even say that a very high potency might hasten death, acting so powerfully. Dr. Close asked whether the evil effects attributed to repeated doses might not sometimes be due to the psychic influence of the prescriber upon the patient. If he fears trouble he will be likely to have it. The prescriber's moods may affect the accuracy of his prescription, and they may also, by operating telepathically, or through what is called personal influence, impress his patient helpfully or otherwise, as well as the remedy prescribed. Dr. Campbell said we speak of moods in developing an art, but the healing art is different. One's moods affect his personality, and an undeveloped art like Homœopathy must be above moods, and will be, with the right conception of art. The scientific curiosity to see what the drug would do had kept her above them. Dr. Close related a case which demonstrated an exception to the usual rule that a high potency should not be followed by a lower. A boy with nocturnal enuresis had for years been treated with high potencies at long intervals without apparent effect. A powder of Pulsatilla$^{200}$, every night for two months, had nearly cured him.

Dr. Fincke gave a case where, after three miscarriages, the patient was threatened once more, and began flooding at the seventh month. Sabina$^{cm}$ was used, and she was worse. Sabina millionth cured in a half-hour. Repetition evidently did it. Also a case of enuresis where the patient slept heavily, Opium$^{90M}$ helped. When given every day it aggravated, but every third night only it acted better.

Dr. Campbell again expressed her longing for a principle to guide one. Dr. Close said the homœopathic remedy is a force or potency to be individualized, and that which influences it must be observed. We cannot determine beforehand how long it will act. The principle Dr. Campbell desires is, repeat when improvement ceases. The doctrine of the single dose includes a repetition of the same dose on the cessation of improvement.

Dr. Campbell asked how we should know when a patient is progressing; by the symptoms, or generally feeling better?

Dr. Close said, if we grade symptoms by their importance, mental symptoms come first, and that would include the "feeling better." Dr. John Campbell gave in diphtheria the Biniode of Mercury$^{cm}$ with no effects, but the 30th four times in water entirely overcame the paralysis of the throat. He also told of a case of metrorrhagia which Sabina$^{9m}$ stopped in three minutes, adding that while he thought he had no mental influence, because of his anxiety, yet he had confidence in the remedy.

Dr. Lutze related a case of sleeplessness with no symptoms except irritability, aggravation from noise, and relief from lying on the stomach. Belladonna$^{cm}$ repeated in a week produced no effect, but the 30th given every morning for two weeks caused sleep at night, which benefit then continued.

Dr. Baylies gave a case of cough and night-sweats and sore sides where Kreosote produced some benefit, but one dose of Bacillinum$^{50m}$ caused the patient to gain four pounds in a week. Also a case of peritonitis with appendicitis in a woman over sixty. The attending symptoms pointed to Pulsatilla. 45M helped, but CM completed the cure.

Dr. Campbell told of the sudden death of her brother from heart trouble, accompanied by attacks of so-called indigestion.

The peculiar feature was the absence of abnormal heart sounds. Puls. and Carbo-veg. had kept the trouble subdued.

Dr. Lutze thought abnormal sounds were not always an accompaniment of valvular lesions, and Dr. Fincke spoke of a man over seventy who had all kinds of noises in his heart.

According to the alphabetical arrangement Dr. Alice Campbell was elected chairman for the next month, and the meeting then adjourned. E. L. CLOSE, *Secretary.*

