# Bacillinum 50m—A Correction

**Published:** 1896-09

**Authors:** B. L. B. Baylies

**Affiliations:** 418 Putnam Ave., Brooklyn


### BACILLINUM$^{50M}$—A CORRECTION.

418 Putnam Ave., Brooklyn, June 12th, 1896.

Editor of The Homœopathic Physician :

I will be much obliged if you will insert in your next number of The Homœopathic Physician the following correction :

The statement of the report of discussion at the Brooklyn

Hahnemannian Union on Repetition of the Dose, in the June number of THE HOMŒOPATHIC PHYSICIAN, at page 270, that but one dose of Bacillin$^{50m}$ given by me to a case of "cough and night-sweats," "caused" the patient to "gain four pounds weight in one week," is an error as to three points, viz.: First, as to the number of doses of Bacillin, not mentioned in my remarks; second, as to time; third, as to fact, that Basillin$^{50m}$ "caused" the gain in weight. I stated that the patient took Bacillin$^{50m}$, not that he took but one dose, and that he reported a gain of four pounds, not limiting the time to one week.

In reply to a question, I stated that the patient visited me weekly, hence the possible misapprehension. The failure on my part to correct the minutes allowed the publication of the error. Very truly yours,

B. L. B. BAYLIES.

